# Hospital and Institutional News

**Published:** 1920-05-08

**Authors:** 


### HOSPITAL AND INSTITUTIONAL NEWS.

### THE WORKER'S NEWSPAPER.

WHEN its founder first established this journal he called it THE HOSPITAL. These two words, he held, covered everything; but, lest their condensation should be too perfect, a sub-title was added to explain them. The various changes which have taken place in the terms of the latter represent nothing but the founder's watchful endeavour to make the definition more and more exact. THE HOSPITAL is the "workers' newspaper," because it is the workers in the hospital world who have always united to produce it.

### MEDICAL AND ALLIED SOCIETIES.

WE again draw attention to the necessity for closer co-operation in our profession and to the fact that the various organisations hitherto at work are able to express the views of their own members alone. A call for the amending of this unsatisfactory situation has been answered by the Federation of Medical and Allied Societies, which aims at uniting and strengthening all existing bodies and is in *competition with none of them.* Already the support has been received of over forty organisations, representative of practically every branch of medical practice, and of the allied professions as represented by the British Dental Association, the Pharmaceutical Society of Great Britain, the College of Nursing, Royal British Nurses' Association, and the Incorporated Midwives Institute. Seven medical M.P.s are amongst those already joined as individuals, and the stage has now been reached when effect can be given to one of the original terms of reference by asking for that moral and financial support which can only be gained by a large accession of *individual associates.* The subscription is limited to 10s. 6d., and members of the profession are invited to send associateship applications to the General Secretary, 6 Vere Street, Cavendish Square, London, W.

### AN ESTATE OF A MILLION AND A HALF.

MR. HOWARD MORLEY, who has left over £1,500,000, was a generous supporter of our leading hospitals and general charities during his lifetime, and has remembered a number of them in his will. Amongst the chief bequests is one of £20,000 to the Master, Fellows and scholars of Trinity College, Cambridge, for the benefit of the College, and another is £10,000 to the London Hospital, which can make good use of this sum at the present time. The Church Army, Salvation Army, Y.M.C.A., Y.W.C.A., and many other social organisations are handsomely remembered, and large legacies are left to a number of relatives.

### A RETIREMENT.

ON April 30 Dr. J. Strickland Goodall was presented at the Middlesex Hospital with a canteen of cutlery and table plate subscribed for by past and present students of the hospital and marking their appreciation of the valuable services he has rendered. Owing to demands on his time Dr. Goodall is retiring from the lectureship in Physiology and Biology, which he has held since 1903. He has also been Sub-Dean of the Middlesex Hospital medical school for thirteen years. His successor will be Dr. Swale Vincent from the University of Manitoba.

### NOTIFICATIONS.

WE would call attention to an official order making important additions to the list of diseases notifiable within the area of the London County Council, which came into force on May 1. The diseases concerned are ophthalmia neonatorum, acute encephalitis lethargica, dysentery, trench fever, acute primary pneumonia, and acute influenzal pneumonia.

### SUPPRESSION OF MALARIA.

SINCE our last issue went to press another stirring letter from Sir Ronald Ross has appeared in *The Times* (April 30), in which the amazing absence of national recognition of the pioneer work performed by certain British doctors in combating and clearing out veritable hotbeds of tropical disease, is vividly brought to the reader's notice. It is impossible here to reiterate the various details he gives, and the points he makes, but we should like heartily to agree with him in his plea that, though the Press of this country rightly urges us to apply to our own lands the powers of medical development and "drive," which it notes as characteristic of certain other communities, yet it might reasonably be expected to remember that the way to achieve this ideal is "to recognise the men who are capable of developing and driving."

### DOCTOR'S SALARY DISCUSSED.

To the institutional worker one of the terrors of the municipal hospital is the possibility, or probability, that his salary will sooner or later become the subject of public discussion. Some may think that such a thought may arise from an overdone mental touchiness, but an old-fashioned feeling of delicacy is uppermost when reading of a long discussion as to whether the medical officer of the Norwich Poor-law Infirmary should (as recommended by the House Committee), or should not (as urged by some of the guardians), receive an increase of £100 a year, raising his salary to £500, the remuneration of a person in a higher walk of life, such as that of a miner. It was urged that quite recently the guardians had had to pay important sums for operations done by general practitioners, and the appointment of Dr. Barclay was in fact relieving the ratepayer. In the end the meeting divided and the majority voted for the increase. The members were so exhausted by their debate that they raised the chaplain's salary from £200 to £260 without discussion. In this compliance with the recommendations of the House Committee he was fortunate, although his rate of remuneration does not appear to be on the generous side.

### RETIREMENT OF BETHLEM'S TREASURER.

A family connection of ninety years with Bridewell and Bethlem Hospitals is completed by the retirement of Lieut.-Colonel A. J. Copeland from the office of treasurer. At the quarterly general court Colonel Copeland was presented with a piece of plate and a cheque, subscribed for by his fellow governors. Sir Charles Wakefield, in making the presentation, said that the late treasurer's association with the hospitals dated from 1865, but his father, Alderman William Taylor Copeland, served as a governor as long ago as 1829, and was president from 1861 to 1868. Thus a chapter of continuous and honourable family service had lasted for the greatest part of a century. Colonel Copeland inherited from his father as a sacred trust the care of these hospitals, which were indebted to him for an invaluable collection of gleanings from the court books and other interesting records of the hospitals handed down from the past, records which threw much light upon the history of the two Royal hospitals from their foundation in 1247 through some six centuries. The new treasurer of Bethlem and Bridewell is Mr. Lionel Faudel Phillips, whose father, Sir George Faudel Phillips, was president in recent years. Thus a continued family connection, such as that of Colonel Copeland, is being carried on.

### VOLUNTARYISM IS THE "HEART BEAT."

Critics inclined to favour the State system will quickly point at once to the war hospitals, State institutions, and will ask what was wrong with those? Lord Knutsford considers that they were not quite the same things as civil hospitals under Government control would be. The whole treatment of our wounded and sick sailors and soldiers would have been very different but for the voluntary assistance of the Red Cross. Those splendid Red Cross workers provided the "heart beat" which made the arrangement human, and which resulted in the whole treatment of the wounded being a part of the war we may well feel proud of. The chairman of "the London" admits freely that the Army Medical Department did splendid work under the most intolerable difficulties. So short, indeed, was the time possible for preparation; on one occasion, for instance, the Director-General was suddenly called upon to supply a full hospital staff and equipment for quite a new campaign, thousands of miles from France, all to be ready in a fortnight! In a State hospital, from the time a patient enters to the time he leaves he will be nothing more than a number. He would be represented by a series of figures, and would fall in a certain category, with an alphabetical indication. No longer would he be a human creature requiring very special consideration and compassion, but merely a "case" for treatment according to a particular page in the official manual, indexed in the papers under A or B. It is a grim picture that Lord Knutsford paints, and he deals with only one aspect of a minor revolution amongst all the revolutions, beneficent and malevolent, that are shaking the nations from top to bottom at the present time.

### SCHOOL CHILDREN TO COME TO THE RESCUE.

We like the idea of the County Council school children coming to the aid of the London hospitals. It is to be brought home to them that the sum necessary to keep things going is a comparatively small matter if it is produced through equally shared generosity on the part of the multitude. If the school child can forgo his Saturday penny (or obtain a second Saturday penny by an appeal to the parent) for a week or two, the £50,000 it is hoped to obtain through the school appeal should be quickly raised. Moreover, the appeal will have an excellent advertising effect in bringing home to the everyday person, which description fits most of us, that his or her hospitals are in danger of departing from the voluntary system that has always so suited the English character. The organisation of the fund will be in the hands of a committee of teachers. There will be practically no expenses, and the Comptroller of the London County Council will act as treasurer. Each teacher will be free to make his appeal and arrange his school collections in the way which to him seems best. So long as the invitation to give is not too much of what a belligerent Irishman called "a cordial invitation," we have nothing but praise to offer in respect of the movement.

### PENSIONS FOR UNEMPLOYABLE BLIND.

Mr. Lloyd George was very frank when he said plainly to the deputation of the blind marchers that the Government could not afford Mr. Ben Tillett's Bill for the alleviation of the lot of the blind. It is an excellent thing that somebody has realised at last that it is impossible for everybody to have everything they need immediately they express a wish for it. A fair amount has been done for the blind in recent years; those unhappily blinded in the war have been looked after very well indeed, which was the least that the nation could do for them. Their lot had the excellent effect of directing public attention to the blind who had never emerged from civilian life. But the Premier was not altogether discouraging to the blind deputation; he told them of the Bill to be brought forward by the Minister of Health, under which conditions it is sure to have a very reasonable chance of leading to legislation. The proposals of the Government, Mr. Lloyd George claimed, were a great advance on anything that had been done in any country in the world. The Bill provides, *inter alia*, that blind persons who are unable to work shall be entitled to a State pension of 10s. per week between the ages of fifty and seventy, on the same conditions as old-age pensions. This provision will cost £137,000 a year.

### STATE OR VOLUNTARY HOSPITALS?

Mr. M'Adam Eccles gave a striking picture of German State-managed hospitals before the war. He found "a veritable parsimony in the working of the hospital, and a great lack of the milk of human kindness. Nothing but bare necessaries anywhere; all these necessaries, I freely admit, are good of their kind; a conspicuous absence of a full and efficient nursing staff, two only, for instance, in a ward occupied by twenty-eight children, a hospital of 600 beds, with some fifty nurses." Lord Knutsford, in a recent talk with a newspaper man, says that a London hospital with 600 beds would have 344 nurses. He thinks that in a State-controlled hospital we should find, very probably, nurses like Post Office clerks, and doctors like Income Tax officials. These people are doubtless very efficient and well suited to the restricted tasks they have to perform, but they are products of State control, and are utterly foreign to what is expected of and gladly given by those who tend the sick and injured.

### THE MAYOR OF NEWPORT'S SUCCESS.

The Mayor of Newport, Councillor Peter Wright, is meeting success with his lightning campaign for funds for the Royal Gwent Hospital. Among the subscriptions he received last week was one of £5,000 from a Cardiff friend. A local colliery proprietor, Mr. Christopher Pond, has given £500 to endow a cot in the name of his grand-daughter, the third cot so endowed by Mr. Pond, who also subscribed £1,000 to the hospital in memory of his wife.

### A MUNICIPAL MATERNITY HOSPITAL.

A Ministry of Health inquiry has been held into the Chesterfield Corporation's maternity and children's hospital scheme. There was no opposition, so the proposal will probably receive official sanction. With the nearest maternity hospital at Sheffield, no one can gainsay the need for the institution, the estimated cost of which is £32,000. It is to be built on a site adjoining the Royal Hospital, the authorities of which have undertaken to work it on an agreed basis of cost. There will be nine beds (not too many for a population of 60,000), with an additional four reserved under arrangement for patients sent by the Derbyshire County Council. For paying patients there will be provided three private wards, and twenty cots for children under five requiring treatment. The cost of maintenance is estimated at £4,000 per annum, and a charge of 4½d. to 5d. in the £ on the rates is involved.

### PATIENTS' CONTRIBUTIONS AT LEAMINGTON.

The governors of Warneford Hospital, Leamington, have decided to adopt the principle of patients' payments; the contributions, we understand, will be voluntary, and based upon the patients' means. Hitherto—for instance, last year—the proportion of patients who contributed toward the cost of their treatment was 3 per cent.

### SUCCESSFUL APPEALS IN WALES.

We are glad to see that over £110,000 has been subscribed to the appeal on behalf of King Edward VII. Hospital, Cardiff. This leaves less than £40,000 to be raised to complete the required sum. At the same time the appeal of the Mayor of Newport for £200,000 on behalf of the Royal Gwent Hospital has already evoked £16,000. In each case steady progress has been made. It ought to put Londoners to shame.

### A BIG PROPOSAL AT ABERDEEN.

A preliminary conference has been held at Aberdeen by the authorities of the Aberdeen Hospitals and of the University to consider the erection of new buildings for the Children's Hospital on a fresh site, the extension of the infirmary, further provision for tuberculous cases by the Town Council, and the medical and scientific needs of the University. A further conference will be held to decide whether it is possible to concentrate the hospitals upon a common site, and to transfer to this site the University departments associated with clinical work. It is argued by the medical profession that the extension of the hospitals on their present premises will not meet the needs of the case, and that a new site can be found within convenient distance of the city. In support of this view, it will be remembered that there is already a scheme to rebuild the Royal Hospital for Sick Children, though the site proposed at Ashley is limited, and another will have to be chosen if the plan for a hospital city is to be carried out.

### THE BLIND IN BUSINESS.

It is unfortunate that the work of St. Dunstan's continues to grow, because of the number of men whom blindness has overtaken since their discharge from hospital apparently recovered from damaged sight. In point of fact the number of men discharged from the Army with damaged sight was 23,000, and consequently it is not easy to estimate the number of relapses which may yet occur. In other respects the record is satisfactory, and the latest annual report is largely filled with letters from men recounting their success in the occupations which they have entered on their discharge from St. Dunstan's. The after-care system of visitors, wholesale purchases of raw materials, a guaranteed central, in the absence of a local, market works well and gives the men confidence to begin with and help in an emergency. Such help does not seem to be often needed, partly because the system exists to forestall emergencies and to prevent mistakes. It is astonishing how much has been accomplished, and Sir Arthur Pearson and his staff deserve the gratitude of the sighted no less than of the blind.

### A BERMONDSEY FIASCO.

The recent sensational accounts of the alleged death by starvation of a young man and his wife were shown to be incorrect at the inquest. The statement was that the man had repeatedly applied to the relieving officer for help, as he was out of work, but had been refused on the ground that both he and his wife were fit to work, and that all that could be offered them was an admission order for the workhouse with their boy, four years old. Only once was an application made for assistance, and then food to the value of 5s. 8d. was given. After that the man went to work for some time, earning 16s. a day. When they were ill an application was made to the relieving officer for the attendance of a doctor, and within two hours of the request father, mother and child were admitted to the Bermondsey and Rotherhithe Hospital. The cause of death in both cases was lobar pneumonia, and it was evident from their condition when they entered the hospital that there was very little hope for them. The woman also gave birth to a still-born child. Not only did the inquest expose the baseless statements that were made, but it exonerated the officer and the board. It is strange, however, that Bermondsey Board of Guardians has not taken advantage of Regulation 12 of the Relief Order to give outdoor relief to cases of able-bodied men with families, out of work. In Southwark nearly twenty families have been assisted in this way, and in every case the Ministry of Health has sanctioned the assistance.

### A FINE START AT MAIDSTONE.

There is no doubt about it that the hospitals of the Provinces are setting the pace for the hospitals of the Metropolis and are, in places throughout the country, nurturing an enthusiasm for hospital work which for the moment is languishing in London. At Maidstone an excellent start has been made with the £100,000 appeal for the West Kent Hospital Enlargement Fund. The hospital week—six days devoted to a veritable hurricane campaign to raise £50,000, the first half of the objective—has by all accounts been a splendid success, and the latest reports speak of at least £40,000 being assured. In accordance with a pledge given in the name of the workers of the town at a meeting at the Concert Hall, a total of £20,000 was promised, to be raised in contributions of 1s. a week for fifty-two weeks from the members of a movement called the Hospital Pay Day Fund. Our workers have brought it home to us very forcibly lately that they have their privileges; it is very grateful to all interested in our hospitals to have such a striking testimony that they realise they have also their responsibilities.

### "ALL PESSIMISTS WILL BE SHOT."

General Seely delivered a stimulating speech at the annual meeting of the Governors of the Royal National Hospital for Consumption, Ventnor, held at the residence of Lord Blyth, in Portland Place. He told the audience how quite recently he was motoring across the Somme battlefield, a place of many sad but glorious memories, when he saw on an old post lying at the foot of a trench the inscription, "All pessimists will be shot." Everything else in that shattered region had disappeared, but this motto, strangely enough, survived. The General rightly thought that the motto was well suited for use in hospital circles at the present time, and he proceeded to speak of the immense value of optimism and hope in combating disease. Mr. P. B. Burgoyne, Chairman of the Board, was justified in his pride in the statement that whereas the expenditure of some other institutions had increased over 100 per cent., by economy at Ventnor the increase had been restricted to 57 per cent. The medical report showed that of the 710 patients treated in 1919 over 78 per cent. showed improvement and 291 were discharged fit for work. During fifty years of work at Ventnor it was probable that the sufferings of a million human beings had been alleviated. An appeal is now made to erect a permanent nurses' home.

### PROFESSIONAL DUALITIES.

The new appointment to the Bishopric Suffragan of Grantham, which recently became vacant through the resignation of Dr. Welbore MacCarthy, marks another of the instances of the close relationship between medicine and the Church, for Dr. J. E. Hine, who has been selected to fill the office, is not only a Doctor of Divinity, but also, previous to his ordination, took his M.D. degree after working at University College, London. Another instance of a combination of professions in one person may be mentioned. It may perhaps not be generally known that the writer of the present-time popular play "The Grain of Mustard Seed" comes from the medical world: Mr. H. M. Harwood having had at one time a resident appointment at St. Thomas's Hospital—in fact it was recently stated that when holding that position he wrote the famous "Theodore and Co." "Lord Richard in the Pantry" is another successful comedy of the day by a medical author; for "Master Swayne" is a pseudonym of Dr. Maurice Nicoll, the son of Sir W. R. Nicoll.

### AN EXTRAORDINARY BEQUEST.

Those of Great Tey in Essex who may be in need of medical and nursing aid are benefited to a considerable extent, writes a correspondent, by the will of the late Major Tudor Lay, who left some £6,000 for that purpose, but the condition that no hospital or nursing home is to derive any benefit directly or indirectly from the bequest is most remarkable! It will be interesting to see the plan adopted for the administration of the legacy.

### THIS WEEK'S DRUG MARKET.

The depressed state of affairs in the drug and chemical markets is still in evidence, and the downward tendency of prices has not been checked. The movement towards lower rates, however, is only pronounced in a few isolated cases, although the general tone of the market may be described as weak. The almost universal opinion among buyers is that the downward movement will not only continue but will become more general, and consequently they are disposed to limit their purchases to their immediate requirements. It is, of course, possible that their opinion is an erroneous one, and there may be a reaction in due course, just as there was shortly after the downward movement which followed the Armistice. Cod-liver oil is one of the articles which buyers are disposed to leave alone for the time being. Menthol, camphor, and dementholised peppermint oil, like other Japanese produce, continue to be in a dull state. Tartaric acid and citric acid are inclined to move downwards in price. Bromides are neglected, and the potassium salt is offered at lower rates. Cloves and clove oil are cheaper.

